# Conversion of Black Carbon Emitted from Diesel-Powered Merchant Ships to Novel Conductive Carbon Black as Anodic Material for Lithium Ion Batteries

**DOI:** 10.3390/nano9091280

**Published:** 2019-09-07

**Authors:** Jae-Hyuk Choi, Dae-Yeong Kim, Won-Ju Lee, Jun Kang

**Affiliations:** Division of Marine System Engineering, Korea Maritime and Ocean University, Busan 49112, Korea (J.-H.C.) (D.-Y.K.)

**Keywords:** black carbon, lithium ion batteries, merchant ships, diesel, conductive, carbon clack

## Abstract

Waste soot generated from diesel engine of merchant ships has ≥ 2 µm agglomerates consisting of 30–50 nm spherical particles, whose morphology is identical to that of carbon black (CB) used in many industrial applications. In this study, we crystallized waste soot by heat treatment to transform it into a unique completely graphitic nano-onion structure, which is considerably different from that of commercial conductive CB. While commercial CB has a large specific surface area because of many surface micropores generated due to quenching by water-spraying in the production process, the heat-treated waste soot has a smooth micropore-free surface. Thus, the treated waste soot acquires the shape of CB but has a much smaller specific surface area. When the treated soot is used as a conductive material in lithium ion battery (LIB) half cells, the Coulombic efficiency of the entire anode is improved significantly owing to its low specific surface area; the electrochemical performance of the LIB is considerably enhanced compared to that of conventional conductive materials. Thus, polluting soot generated in marine propulsion can be transformed into a new class of CB with a unique structure by simple heat treatment; this soot can also be used as an inexpensive conductive material to enhance the LIB performance.

## 1. Introduction

Black carbon (BC) is a solid particulate material produced by incomplete combustion of carbon-based fuels. It generally contains 80% mass or more of carbon atoms that are usually connected by sp^2^ bonds. BC from marine diesel engines is estimated to account for as high as 1–2% of the world’s total BC generation [1]. This is because the ship fuel is lower in quality than other fossil fuels used in combustion engines, and therefore more BC is emitted per unit of ship fuel consumed [2].

While global warming is a growing concern worldwide, the Arctic climate is known to be warming up almost twice as fast as the rest of the world [3,4]. BC is responsible for half of the Arctic warming [5], as it strongly absorbs incoming and reflected light irradiation [6,7] and accelerates melting of snow and ice when it is deposited on these surface [8]. Further, as a result of the shrinking Arctic ice, the Arctic shipping route has begun to develop, and an increase in shipping through that region will lead to even more BC discharge/deposition, thus exacerbating the warming.

In 2005, the International Maritime Organization (IMO) adopted the International Convention for the Prevention of Marine Pollution by Maritime Pollution Prevention Commission (MARPOL 73/78) in order to limit the emission of various air pollutants (NOx, SOx, and volatile organic compounds (VOC)). The regulation requires that all ocean vessels around the world use fuels with a sulfur content below 0.5% by the year 2020 [9]. To address such regulations, shipping companies have considered three approaches: (1) use of low-sulfur oil, (2) use of liquefied natural gas (LNG) fuel, and (3) installation of scrubbers. Option (1) is the most viable in the short term, but the operating cost is high in the long run. The adoption of LNG fuel entails a high financial investment to construct LNG facilities, since currently the LNG fuel is not convenient owing to insufficient supply facilities around the world [10]. Moreover, LNG prices tend to fluctuate unpredictably, and the problem associated with methane emission remains. In contrast, usage of scrubbers involves a high cost of installation; however, they can be applied directly to existing ships. Thus, many shipping companies are using this approach because it allows the continued usage of high-sulfur oil [11]. As the number of vessels equipped with these scrubbers increases, the amount of BC discharged from the vessel will be reduced.

Scrubbers are divided into two types depending on the operation method. The loop-type scrubber circulates the washing water used to remove air pollutants from the ship emission, while the open-type discharges it into the sea. At present, regulations related to the discharge of washing water are insufficient. Therefore, many ship owners prefer the open-type scrubber because they are relatively inexpensive and easy to operate, even though they may be restricted through future regulations. To ameliorate the considerable marine pollution caused by BC collected from ship scrubbers, in this study, we attempted to reuse BC as a valuable material in electrochemical applications.

First, we confirmed that the morphology of the collected BC particles is similar to that of carbon black. In general, carbon black is produced through incomplete combustion or pyrolysis of hydrocarbons such as petroleum or natural gas; it is characterized by chain aggregates at least 1 μm long formed by primary particles that are 10 to 30 nm in size [12]. Owing to the nature of the starting material, carbon black generally contains more than 95% net carbon and a minimum amount of oxygen, hydrogen, and nitrogen. The particle size, agglomerate size, porosity, and surface chemical properties of carbon black can be controlled through various process parameters. Based on these unique properties, carbon black is often used as an additive to improve the physical, electrical, electrochemical and optical properties of materials. For example, it is used at a high volume fraction as a reinforcing and performance additive in rubber products. The excellent conductivity of carbon black also makes it an electrode material for energy storage media. In the printing industry, it is used as a viscosity control additive for pigments and optimum print quality. As an additive, it may improve the performance of other materials, including electrostatic charge control and ultraviolet shielding [13,14,15,16,17,18,19,20,21].

Owing to the identical morphology, BC from ships could be used in the above applications. Among them, a very attractive application would be reusing BC, a pollutant generated from the power source that moves the ship (i.e., the combustion engine) as an energy material in batteries to move the ship. Importantly, crystallization through an additional heat treatment process can increase the conductivity of the waste BC and make it a better conductive material in the lithium ion battery (LIB). We confirmed that the heat-treated BC has significantly lower surface area compared to conventional commercial carbon black, and it could substantially improve the Coulombic efficiency of the anode material. In addition, BC is a waste material produced by ships, while commercial carbon black tends to consume hydrocarbon as feedstock. Therefore, BC can be very attractive in terms of cost.

## 2. Materials and Methods

### 2.1. Sampling

**Figure 1:**
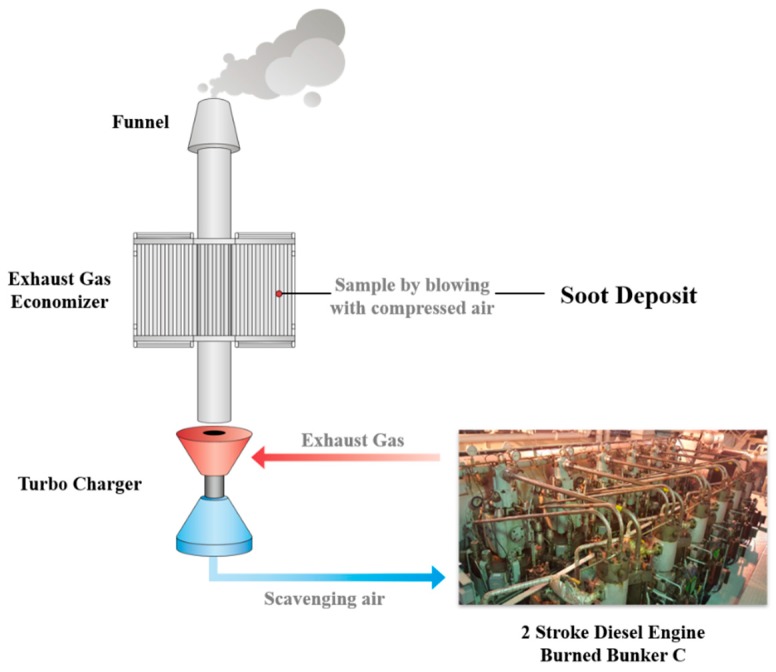
Schematic of soot sampling.

Waste soot was collected from a container ship operated by Korea Leading Company of Ship Management Co., Ltd. (KLCSM). Table 1 shows the detailed specifications of the ship and its diesel engine. In the ship’s engine room, a large amount of waste soot is generated in various machines when using Bunker C as fuel oil. In this study, we collected waste soot from the economizer, which accumulates the largest amount of soot. The economizer is a kind of heat exchanger used to boil water by recycling the waste heat in hot exhaust gas from the ship’s main engine. Before the exhaust gas is passed through this machine and vented into the atmosphere, a large amount of waste soot in it will be deposited on the heat exchanger pins. The specifications of the ship and the schematic of the economizer are shown in Table 1 and Figure 1, respectively. Details about the bunker fuel oil are given in Table 2, and those of the engine are given in Table 3.

### 2.2. Heat Treatment Procedure

For crystallization and purification, the waste soot was heat-treated at four different target temperatures in an ultra-high-temperature furnace (Thermvac Engineering, Gyeonggi-do, Korea). For the samples labeled A and B, the soot was heated at 7 °C/min to the respective target temperatures of 1400 °C and 1700 °C under Ar atmosphere. Sample C was first heated to ~1800 °C at 10 °C/min, then to ~2300 °C at 5 °C/min. Sample D was heated in three stages: first to ~1800 °C at 10 °C/min, then to ~2400 °C at 5 °C/min, and finally to ~2700 °C at 3 °C/min. After reaching the target temperature, all samples were maintained for 5 h and then cooled naturally to ambient temperature.

### 2.3. Soot Characterization

The shape and microstructure of the waste soot was observed by transmission electron microscopy (TEM, JEM-2100F, JEOL, Akishima, Tokyo, Japan) at an accelerating voltage of 200 kV. The specific surface area of soot was determined from the N_2_ adsorption-desorption isotherms obtained using a Quantachrome sorption analyzer (Autosorb-1, Boynton Beach, FL, USA) by the Brunauer–Emmett–Teller (BET) method. Before the N_2_ adsorption-desorption experiments, all samples were heated under N_2_ at 200 °C for 2 h to remove moisture. Thermogravimetric analysis (TGA) was performed using a TGA Q500 device (TA Instrument, New Castle, DE, USA) to analyze the weight of the soot residue. To obtain the elemental composition, a carbon-hydrogen-nitrogen-sulfur (CHNS) analyzer (Thermo Fisher Scientific, EA1112, Waltham, MA, USA) was used.

### 2.4. Electrochemical Measurement

Electrochemical testing was performed using a 2032-coin type (Wellcos Corp., Gunpo-si, Korea) half-cell. The slurry for electrode fabrication was prepared by dissolving the active material (artificial graphite powder, specific surface area ≤4.2 m^2^/g, MTI Korea, Seoul, Korea) (70 wt%), heat-treated soot (10 wt%) as the conductive material, and a binder (polyacrylic acid, average molecular weight 3,000,000) (20 wt%) in distilled water. The prepared slurry was coated on a Cu-foil substrate using a doctor blade coater, and then dried at 50 °C for 12 h to remove the solvent (The copper foil was dried at atmospheric pressure for 10 h and the last two hours in a vacuum atmosphere). It was then pressed down to a thickness of 35 μm (thickness of the active material only) by a roll press. CR2032-type coin cells were fabricated in a glove box filled with Ar using lithium coin chips as the counter electrode and the reference electrode, Celgard 2400 as a separator, and 1 M LiPF_6_ in ethylene carbonate (EC)/diethyl carbonate (DEC) (1:1, *v*/*v*) containing 10 wt% fluoroethylene carbonate (FEC) as the electrolyte. Electrochemical testing was performed using a BCS-805 battery test system (Biologic, Seyssinet-Pariset, France) in the voltage range of 0.005–3 V (vs. Li/Li^+^). The same device was used to perform cyclic voltammetry (CV) to investigate the reduction and oxidation peaks in the voltage range of 0.01–3.0 V (vs. Li/Li^+^) at a scan rate of 0.2 mV/s.

## 3. Results

**Figure 2:**
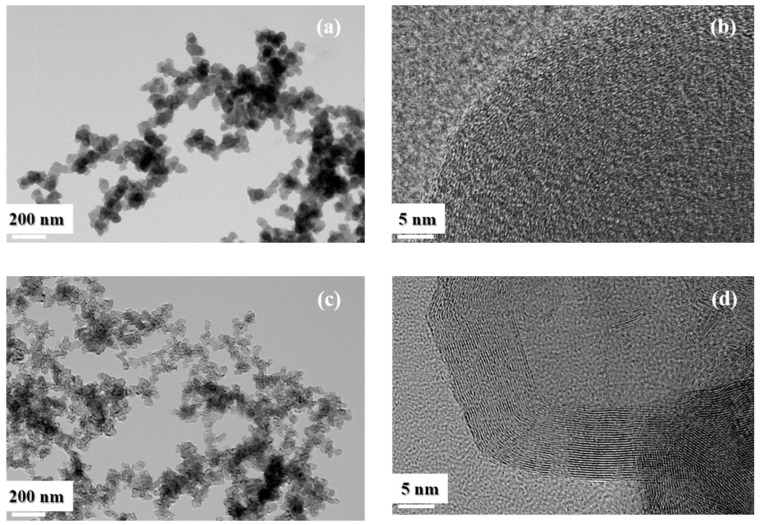
TEM images of (**a**) as-obtained soot, (**c**) annealed soot. (**b**,**d**) are the corresponding HR-TEM images.

Figure 2a shows a TEM image of the soot collected from the economizer. The observed morphology confirms that the primary particles are spherical and approximately 30–50 nm in size, and that these particles are aggregated into chain structures. The high-level agglomerate structures are large and generally longer than 2 μm. Since such a structure can easily form an intergranular network over a wide scale, it is expected to provide excellent conduction between the active material particles in the battery electrode. The high-resolution (HR) TEM image of the untreated soot (Figure 2b) shows an amorphous structure without crystalline domains. During the high-temperature heat treatment at 2700 °C, crystallization proceeds from the outside of the spherical particle to the interior part (Figure 2c,d). Spherical primary particles have been converted into angular spheres, some of which contain hollow cores.

Meanwhile, in the HR-TEM image, the commercial carbon black (Figure S1) shows a considerably different shape than that of waste soot (Figure 2d). Based on electron microscopy studies, a number of models have been proposed for the structure of common commercial carbon black particles. Most of them describe the carbon particles as spherical arrays of quasi-microcrystalline domains, which are more disordered at the center as the size becomes smaller.

X-ray diffraction studies reveal that the commercial carbon black has a large *d*-spacing (3.5–3.6 Å) compared to graphite carbon (3.354 Å). The quasi-graphite layers are oriented parallel to each other, but they are turbostratic. These microcrystalline domains have been reported to have only a few layers and a width of approximately 20–30 Å. TEM images of the actual conductive carbon black (Figure S1) also support this interpretation. Thus, commercial carbon black can be said to have many microcrystalline domains that aggregate into the primary particles, which then form lumps. In contrast, waste soot particles show a smooth spherical shape without the fine domains. After heat treatment, their structure turns into an onion-like structure with a smoother surface, and such morphology changes the material properties significantly. In particular, in commercial carbon black, micropores exist between the microcrystalline domains, owing to which and it has a very large specific surface area, while the heat-treated waste soot is expected to contain no micropores owing to the long and continuous crystallization.

**Figure 3:**
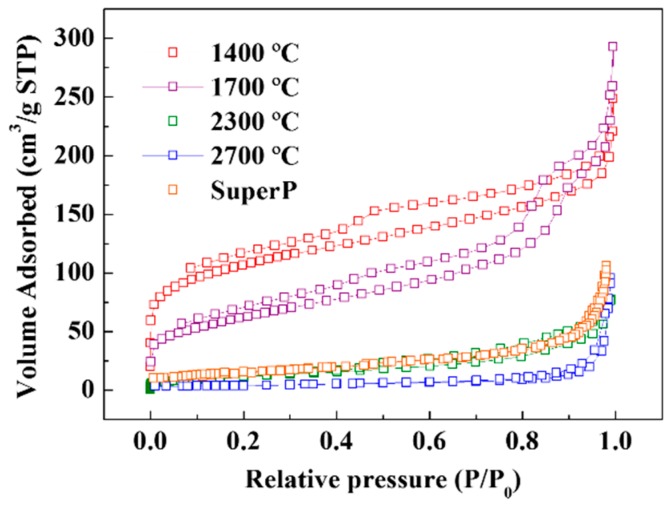
Nitrogen adsorption-desorption isotherms of annealed soot and SuperP.

The difference in the specific surface area was confirmed by the BET analysis. Figure 3 shows the N_2_ adsorption isotherms of waste soot according to the heat treatment temperature along with that of SuperP (a commercial carbon black). Table 4 shows the specific surface area calculated from the isotherms. The specific surface area of SuperP is high, above 50 m^2^/g, and that of untreated waste soot is also relatively high, owing to its amorphous structure, as predicted by the TEM results. However, as the heat treatment progresses, the particle structure changes to a crystalline onion structure, and the final specific surface area is very low (less than 10 m^2^/g). Such a low value indicates the absence of micropores on the surface, thus supporting the TEM observation.

To the best of our knowledge, materials with morphology identical to that of carbon black but with a specific surface area below 10 m^2^/g have not been reported to date. Therefore, this heat-treated soot was expected to possess unique properties. While the low specific surface area makes it unsuitable as a catalyst support, as it improves the Coulombic efficiency, the heat-treated soot is more advantageous compared to commercial conductive materials for use in LIBs. Therefore, we hypothesized that the heat-treated soot can be used as a conductive material in a LIB to increase the Coulombic efficiency of the entire electrode.

**Figure 4:**
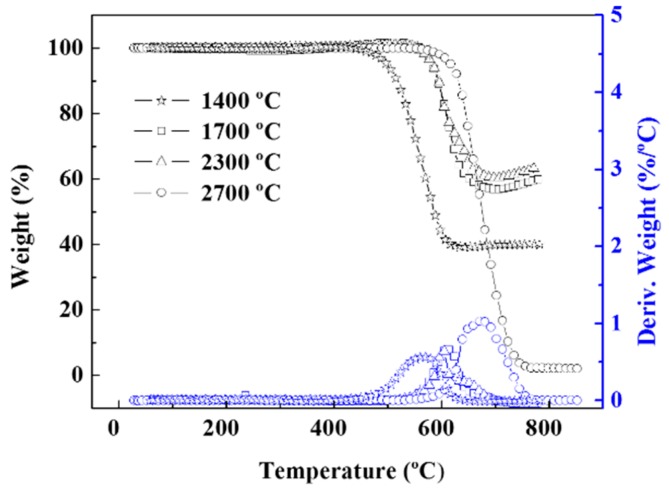
Thermogravimetric analysis graph of annealed soot.

In the electrodes, various impurities in the waste soot may affect the electrochemical performance. Therefore, CHNS elemental analysis (Table 5) and TGA thermogravimetric analysis (Figure 4, Table 6) were used to quantify these impurities.

The CHNS results confirmed that considerable amounts of sulfur and hydrogen existed in the soot before heat treatment. Since the soot is a combustion product of high-sulfur oil, it is likely to contain a large amount of sulfur. However, as the heat treatment temperature increased, the amount of sulfur and hydrogen decreased sharply and completely disappeared at 2700 °C.

As can be seen from the CHNS results, the carbon composition ratio increases as the heat treatment temperature increases. Thus, soot heat-treated at very high temperatures consists of only carbon. Because carbon is generally gasified by combining with oxygen of ~500–700 °C, it is oxidized during TGA analysis and disappears. Therefore, for soot heat-treated at 2700 °C, typically, no residue is observed during TGA analysis. On the other hand, the main component of the residue is a metal oxide [2,22,23,24]. The low-quality heavy fuel oil is used as a fuel oil for ships, which contains a significant amount of organometallic compounds (particularly, vanadium), and sometimes metal oxide catalysts are used to crack the crude oil. Meanwhile, these compounds do not decompose at low temperatures, but only at very high temperatures; therefore, the residues are considerably reduced in soot heat-treated at 2700 °C.

**Figure 5:**
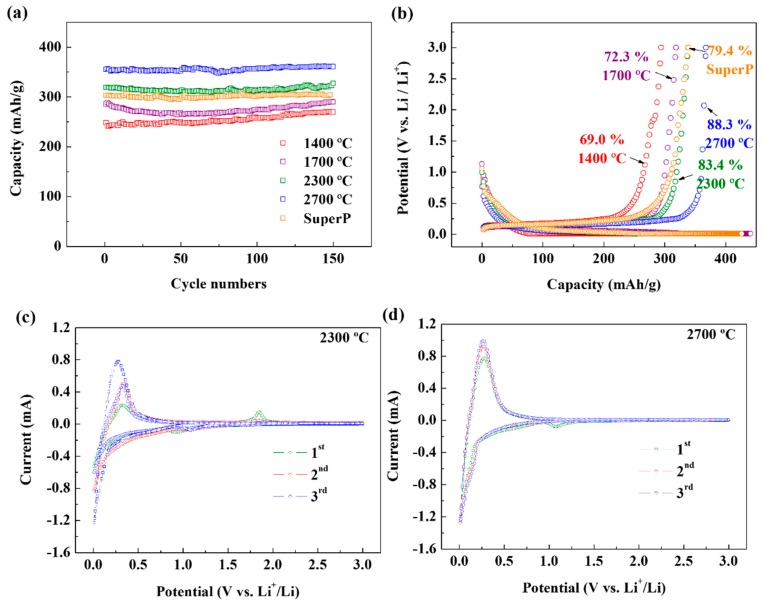

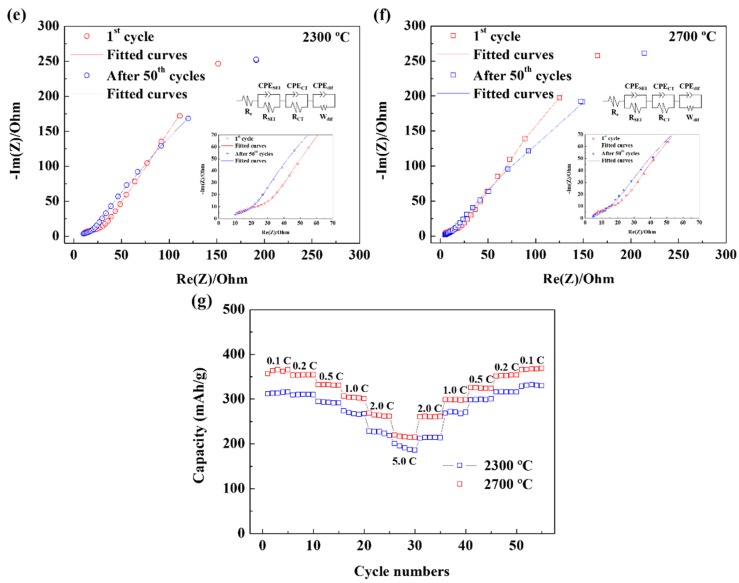
(**a**) Cycling performance of the annealed soot and SuperP at a rate of 1 C. (**b**) Discharge/charge profiles for the first cycle at a rate of 0.2 C. The numbers indicate the initial Coulombic efficiencies. (**c**) CV curves of the annealed soot for the first cycle at a scanning rate of 0.2 mV/s in voltage range of 0.005–3 V (vs. Li/Li^+^). (**d**) CV curves of soot annealed at 2700 °C at a scanning rate of 0.2 mV/s in voltage range of 0.005–3 V (vs. Li/Li^+^). (**e**,**f**) Nyquist plots of the soot annealed at 2300 and 2700 °C after 1 and 50 cycles. (**g**) Rate capability for soot annealed at 2300 and 2700 °C.

A galvanostatic charge/discharge experiment was performed to evaluate the electrochemical performance of the heat-treated soot as a conductive material in the anode. Figure 5a shows the reversible capacity data according to the cycling of the electrode.

The soot annealed at 1400 °C showed the lowest reversible capacity, while that annealed at 2300 °C showed a capacity comparable to that of SuperP. Moreover, the first-cycle Coulombic efficiency shows that the soot treated at 2300 °C exceeds SuperP in efficiency (Figure 5b). Since heat-treated soot has a much smaller specific surface area than SuperP, a lower amount of the solid electrolyte interphase (SEI) layer is formed on its surface, and consequently, the Coulombic efficiency of the entire electrode material is increased. Therefore, in order for the soot to surpass commercial carbon black in performance as a conductive material, the crystallization should be carried out at a temperature above 2000 °C. Figure 5c compares the CV curves of the first cycle for the annealed soot samples, confirming the presence of residual impurities in a voltage range of 0.005 to 3 V. For the soot annealed at 1400 °C, redox peaks, which are presumed to be due to impurities in the carbon rather than electrolyte decomposition, appeared at 1.8 V. However, for soot annealed at 2700 °C, these impurity peaks disappeared. The first three cycles of the CV curves for the soot annealed at 2700 °C (Figure 5d) confirm the formation of a stable SEI layer. The first peak located at 1.1 V can be assigned to the irreversible reduction of the electrolyte additive FEC. The second broader cathode peak at 0.25–1.1 V corresponds to the decomposition of EC/DEC and the formation of an SEI layer. Finally, a sharp peak indicating the insertion of Li ions is observed at 0.25 V or lower. After the first cycle, the cathode reduction peaks disappear, and CV curves in different cycles almost overlap without any apparent change in the peak current or peak potential. This indicates the excellent reversibility of the Li insertion and extraction reactions and the stability of the soot-based conductive material.

Impedance measurements were performed to examine the degradation of the soot-based conductive material after numerous cycles. Figure 5e,f show the experimental data after the first and 50th cycles. Even after 50 cycles, it maintained the same value of resistance at high frequency, and so the soot-based conductive material between the graphite particles shows stable performance. The parametric values analyzed from EIS are summarized in Table 7.

The C-rate capability of the soot-based conductive material is shown in Figure 5g. The reversible capacity decreased as the C-rate increased from 0.1, 0.2, 0.5, 1, 2, to 5 C. The reversible capacity reduction rate at 0.2 C versus 2.0 C was 76%, indicating excellent C-rate capability. Finally, after cycling at high C-rates, the capacity gradually recovered when the C-rate was decreased: when the current rate returned to 0.1 C in the 55th cycle, the reversible capacity was 367 mAh/g. These results indicate that the heat-treated soot performs reliably at various current densities.

These measurements show that the heat-treated waste soot displays high Coulombic efficiency owing to its low specific surface area, with better electrochemical performance than that of commercial carbon black. Therefore, the heat-treated waste soot can function as a unique conductive material to increase the Coulombic efficiency of secondary batteries. Moreover, we demonstrated that soot emitted from ship engines, which is usually considered a pollutant, can be utilized as a superior electrode material for energy storage after a simple heat treatment. While increasing the amount of global cargo shipping would lead to increased generation of soot in the coming years, we have found a great way to reuse it.

**Figure 6:**
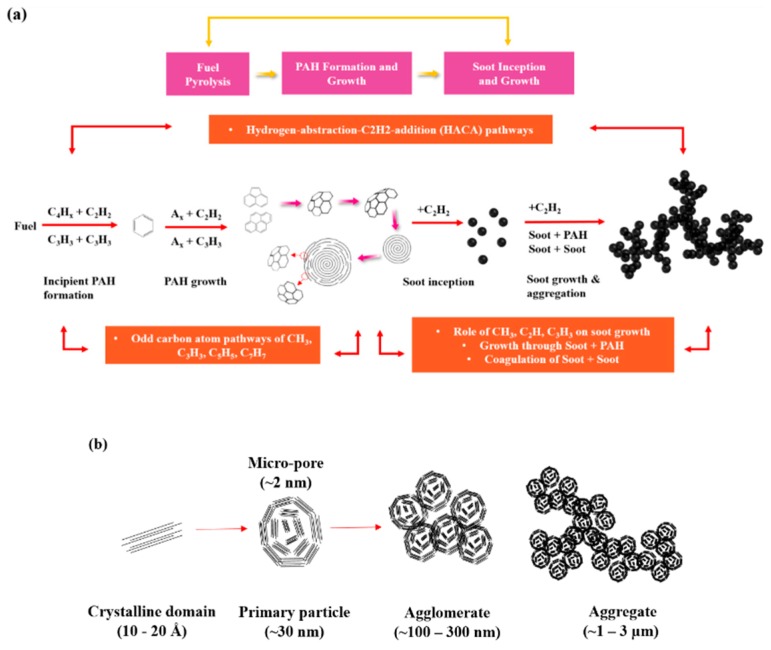

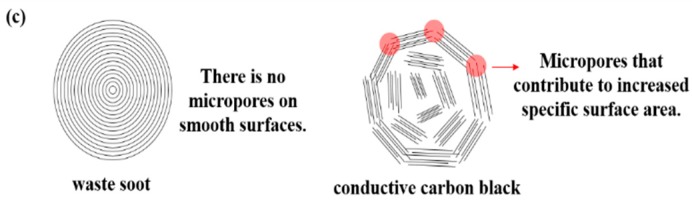
Schematic of the formation mechanism and structure of (**a**) waste soot and (**b**) commercial conductive carbon black. (**c**) The relationship between surface structure and surface area.

Finally, we consider the reasons why waste soot can develop a carbon black structure, and why it grows into smooth spherical particles rather than small microcrystalline domains. This observation can be deduced from the difference between the combustion process in the ship engine and the commercial process of carbon black production (Figure 6).

First, in the engine’s combustion chamber, pyrolysis of the fuel produces odd carbons such as methyl group (CH_3_), propargyl (C_3_H_3_), and benzene (C_6_H_6_) ring by C_2_H_2_. The benzene rings fuse together to form polycyclic aromatic hydrocarbons (PAHs). The addition of C_2_H_2_ leads to much larger PAH molecules, which coalesce to form primary particles several nanometers in size [25,26,27,28,29,30]. At this time, it is unclear how the spheres are generated from large nuclei of carbon atoms/radicals, but one of the most widely proposed hypothesis is their nucleation from a pentagonal carbon ring following spiral shell growth [31,32,33,34]. This can be considered to process in four steps: (a) pentagonal nucleation, (b) growth of quasi-cubic shells, (c) formation of helical shell carbon particles, and (d) growth in size. On the other hand, the primary spherical particles formed through the above process are further grown by the HACA (hydrogen abstraction acetylene addition) reaction in the flame, and coagulation of the primary particles results in high-level structures that flow in the flame. Particularly, in a ship diesel engine, particles generated in the combustion chamber at high-temperature and high-pressure pass through a very long exhaust pipe at ~300 °C, and the temperature gradient therein causes soot coagulation by thermophoresis. In other words, in the exhaust pipe, aggregates formed in the combustion chamber become smooth spherical particles in a continuous agglomeration process at the high temperature. Upon collecting the soot and subjecting it to a crystallization process through high-temperature heat treatment, the particle surface became perfectly smooth with an onion structure, and the particles grew into smooth spherical agglomerate structures without micropores.

In contrast, carbon black often undergoes rapid temperature changes during its production. Specifically, a significant amount of quenching water is directly sprayed several times into the combustion chamber to control the temperature. In addition, quenching water is sprayed several times during the wet palletization, separation, and collection of carbon black. Thus, carbon black often undergoes rapid temperature changes during the growth process, which make it impossible for it to achieve continuous surface growth with PAH. As a result, during the condensation under decreasing temperature, the nanocrystals agglomerate into a number of nanodomain states instead. These nanodomains are presumed to form a porous structure on the surface instead of a perfect spherical structure (Figure 6c).

## 4. Conclusions

We found that waste soot generated from ship engines can be converted to novel and useful energy material through a heat treatment process. During its generation, the soot develops a carbon-black-like morphology as it grows into spherical particles and coagulates under the combustion environment in the internal combustion engine. During the heat treatment, the soot particle surface became very smooth with a nano-onion structure, which is rather different from the porous morphology of commercial carbon black. Owing to the lack of micropores, the total specific surface area of the soot is significantly reduced after heat treatment (to one-tenth that of commercial carbon black). Using the heat-treated soot as a conductive material in LIBs can increase the Coulombic efficiency ‘as like the anodic half cells materials, studied here,’ because of the considerably reduced specific surface area. We consider the heat-treated soot as a new type of carbon black. Although this study only considered LIBs, the soot-based material may be applied in many fields, for e.g., as shielding of electrical cables and special electrical parts in cars, where its unique characteristics are desired. Hence, although waste soot from ships is currently considered an air pollutant, it could be exploited as a valuable material in energy storage and possibly other uses.

## Figures and Tables

**Table 1:** Technical description of the container ship (M/V SUNNY SPRUCE).

Items	Description
Gross Tonnage	3981 Mt
Length Overall	107.3 m
Breath	17.2 m
Maximum Speed	16.78 knots
Engine Model	MAN B&W 7L35MC
Output x RPM (MCR)	5320 PS × 200 RPM
F.O. Consumption (at sea)	HFO: 15.3 MT (at NCR)
Kind of Fuel Oil	IFO 380 cSt

**Table 2:** Engine specifications.

Equipment	Items	Specification
2-stroke diesel engine	Manufacturer	MAN Diesel & Turbo
	Model	7L35MC
	MCR	5320 PS × 200 rpm

**Table 3:** Fuel oil specifications.

Parameters	Unit	Results
Specific gravity @15/4 °C	-	0.9867
Viscosity Kin. @50 °C	mm^2^/s	321.3
Flash point	°C	74
Sulfur content	Weight %	2.89
Water sediment	Volume %	0.05

**Table 4:** Results of N_2_ adsorption/desorption analysis.

Sample	BET (m^2^/g)
A: 1400 °C	376.85
B: 1700 °C	223.38
C: 2300 °C	41.87
D: 2700 °C	13.25
SuperP	53.28

**Table 5:** CHNS elements analysis results of annealed soot at different temperatures (wt%).

Sample	Carbon	Hydrogen	Nitrogen	Sulfur
A: 1400 °C	69.49	0.01	Not detected	29.71
B: 1700 °C	78.29	0.01	Not detected	20.79
C: 2300 °C	87.95	Not detected	Not detected	12.04
D: 2700 °C	98.64	Not detected	Not detected	Not detected

**Table 6:** Thermogravimetric analysis data of annealed soot.

Sample	Initial Decomposition Temperature (°C)	10% Weight Loss Temperature (°C)	Weight Loss (%)	Residue at 850 °C (%)
A: 1400 °C	544.95	556.67	59.96	40.05
B: 1700 °C	629.08	645.39	40.55	59.46
C: 2300 °C	635.35	647.19	36.79	63.20
D: 2700 °C	632.17	632.12	97.86	2.143

**Table 7:** The parameters of EIS measurements for soot annealed at 2300 and 2700 °C.

	R_e_ (Ω)	R_SEI_ (Ω)	R_CT_ (Ω)
2300 °C			
1st cycle	2.213	31.43	53.72
After 50th cycles	2.714	32.59	79.12
2700 °C			
1st cycle	1.849	34.6	36.07
After 50th cycles	2.696	33.14	43.04

## Supplementary Materials

The following are available online at https://www.mdpi.com/2079-4991/9/9/1280/s1, Figure S1: Transmission electron microscopy images of (a) Ketjen black and (b) Vulcan black.
