# Supplementary material for: Conversion of Black Carbon Emitted from Diesel-Powered Merchant Ships to Novel Conductive Carbon Black as Anodic Material for Lithium Ion Batteries
Source: Nanomaterials (Basel). 2019 Sep 7;9(9):1280. doi: 10.3390/nano9091280 (PMC6781079; doi:10.3390/nano9091280)
Supplement: Supplementary file 1 [file nanomaterials-09-01280-s001.pdf]

Supplementary information

Conversion of Black Carbon Emitted from Diesel-Powered Merchant Ships to Novel Conductive Carbon Black for Lithium Ion Batteries

Jae-Hyuk Choi [a], Dae-Yeong Kim [b], Won-Ju Lee [b,*] & Jun Kang [b,*]

[a] Division of Marine System Engineering, Korea Maritime and Ocean University, Busan 49112, Korea

[b] Division of Marine Engineering, Korea Maritime and Ocean University, Busan 49112, Korea

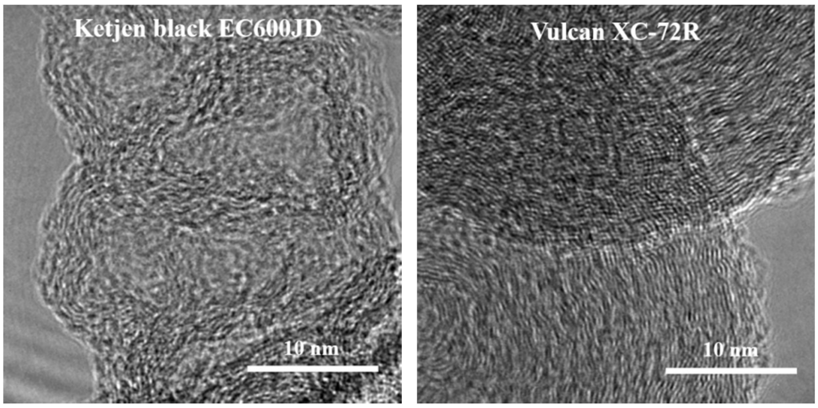

(a) (b)

Fig. S1. Transmission electron microscopy images of (a) Ketjen black and (b) Vulcan black.
